# Effect of accreditation on length of stay in psychiatric inpatients: pre-post accreditation medical record comparison

**DOI:** 10.1186/s13033-016-0090-6

**Published:** 2016-09-07

**Authors:** Mohammed Abdullah Al-Sughayir

**Affiliations:** Psychiatry Department, College of Medicine, King Saud University, PO Box 21525, Riyadh, 11485 Kingdom of Saudi Arabia

**Keywords:** Accreditation, Inpatient, Length of stay, Mental health, Quality, Psychiatry, Saudi Arabia

## Abstract

**Background:**

An interest in hospital accreditation is growing rapidly among many countries to enhance the quality of health care services. The literature showed a positive association between accreditation and some processes of health care. One of the main factors that influence bed availability is the length of hospital stay (LOS), which is considered as an important indicator of the quality of inpatient psychiatric hospitalization. We aimed to investigate whether hospital accreditation drives improvements for the length of stay in psychiatric inpatients.

**Methods:**

The study reviewed medical records of consecutive hospital admissions for pre- and post-accreditation comparisons of LOS in two acute mental health wards at a teaching general hospital in Riyadh, Saudi Arabia. Data obtained from the 12-month-post-accreditation period (July 2011 to June 2012) were compared with those from the 12-month-pre-accreditation period (July 2009 to June 2010). The adoption of accreditation program occurred over a 12-month period in the middle of the study (July 2010 to June 2011). Compiled information included demographics, diagnosis, assessment, and LOS. All identified charts were reviewed; there were no exclusion criteria. Patients were not contacted.

**Results:**

Post-accreditation, the mean (SD) length of stay was 35.3 ± 18.5 days and the range was 3–113 days. Whereas in the pre-accreditation period the mean (SD) length of stay was 41.1 ± 29.5 days and the range was 1–167 days. The difference was statistically significant (P = 0.026).

**Conclusion:**

Accreditation reduces excess LOS and contributes to improving the quality of psychiatric inpatient care and access to psychiatric beds.

## Background

An interest in hospital accreditation is growing rapidly among many countries to enhance the quality of health care services. The literature showed a positive association between accreditation and some processes of health care [1–3]. Admissions at fully accredited hospitals were associated with a shorter LOS compared with admissions at partially accredited hospitals [4]. Accreditation was associated with better outcomes of bariatric surgery [5, 6]. A study evaluated the effect of accreditation on pain management of patients undergoing surgeries showed an overall increase in the average consumption of opiates not associated with an increased length of stay [7].

In the eastern Mediterranean region, Saudi Arabia was one of the first countries to implement health care accreditation standards [8]. Several governmental hospitals in Saudi Arabia have received accreditation from different international certification bodies [9].

Health services researchers consider the length of hospital stay (LOS) as an important indicator of the quality of inpatient psychiatric hospitalization [10]. Since accreditation is a process to enhance the quality of patients’ care, the identification of modifiable determinants of LOS could lead to improvements in the quality of patients’ care. As delayed discharge from hospital creates additional pressure on staff; there is a global tendency to shorten psychiatric hospitalization [11]. Studies of LOS are an essential component of any attempt to describe the operation of psychiatric services.

The average LOS in acute mental health facilities for patients with predominantly positive psychotic symptoms ranged from 10.5 days to 43 days [12–14].

Given the increasing need and limited availability of inpatient psychiatric beds, their optimal use should be a high priority. In Saudi Arabia, the bed occupancy in most psychiatric hospitals is approximately 100 % throughout the year (the ideal figure is 85 %) [15].

Despite numerous studies that focused on LOS, effect of accreditation on psychiatric inpatient LOS has received little attention. Based on the PsychINFO [16] and Medline/PubMed [17] computerized databases, the impact of accreditation on LOS in psychiatric inpatients in Saudi Arabia has never been studied. Thus, this study attempted to investigate whether accreditation drives improvements for LOS in psychiatric inpatients, intending to enhance the mental health care delivery system in the country.

## Methods

### Site

The inpatient psychiatric units at King Khalid University Hospital (KKUH) in Riyadh, Saudi Arabia, which is the only public teaching hospital in Riyadh City with an 800-bed capacity. The psychiatric units comprise 22 mental health beds (11 for each sex) in locked-door wards, and adequately staffed with psychologists and social workers along with medical and nursing personnel. Patient admissions are usually through outpatient clinics, the emergency department, and rarely from medical wards. Hospitalization includes planned diagnostic assessments, stabilization of crisis presentations, and brief intensive treatment.

### Design

The study reviewed medical records of consecutive hospital admissions for pre- and post-accreditation comparisons of LOS in two acute mental health wards.

### Data collection

A structured data collection sheet has been made including the following factors for each patient demographics, diagnosis, assessment, and LOS. The length of stay in hospital (recorded in days) was calculated by subtracting the date of admission from the date of discharge. The psychiatric inpatient case register in each unit was accessed to identify all in-patients admitted and discharged during the two study periods. Data in the case register are recorded by nursing staff under the direct supervision of an expert head nurse. After patient’s discharge, the medical file is forwarded to the Medical Record Department at KKUH. Clinical data were extracted in October 2012 by the author from patients’ files at the Medical Record Department. Data were paper record-based information. The quality of data records was identified and assessed based on the availability and legibility of a detailed documentation for all admissions in both study periods. The psychiatric nurses, under the supervision of the unit head nurse, recorded the required data in legible English. The data were then coded and entered into statistical software.

### Subjects

Psychiatric inpatients admitted during the 12-month-post-accreditation period (July 2011 to June 2012) were compared with those from the 12-month-pre-accreditation period (July 2009 to June 2010). All identified charts were eligible for review including patients leaving against medical advice; there were no exclusion criteria. However, to maintain independence of observations, only the first hospitalization per patient during the study period was included and the charts of patients who were readmitted during the study period (three patients in the pre-accreditation and two patients in the post-accreditation periods) were excluded. Patients were not contacted.

### Intervention

The accreditation process which is a system of strategic planning to promote the quality of the clinical practice. The accreditation program included 18 mental health standards focusing on patients’ safety, biopsychosocial multidisciplinary team (MDT) approach with an objective assessment of symptoms severity through the brief psychiatric rating scale (BPRS), adoption of clinical practice guidelines, rapid evaluation, and clear discharge plan. The standards incorporated the Plan-Do-Check-Act circle. The adoption of accreditation program occurred over a 12-month period (July 2010 to June 2011) in the middle of the study. A team of surveyors examined hospital’s compliance with the accreditation Canada international standards during an onsite survey. Hospital performance was assessed based on reviewing guidelines, interviewing staff, and conducting tracers. Based on these findings, the hospital as a whole was awarded accreditation in February 2011.

### Analysis

The collected data were entered into a spreadsheet for analysis. Statistical analysis was conducted using Statistical Package for the Social Science (SPSS) version 15 software for Windows (SPSS Inc., Chicago, IL, USA). A P value of <0.05 indicated statistical significance.

## Results

There were 182 patients, during the post-accreditation period, compared to 177 patients during the pre-accreditation period. The socio-demographic and clinical characteristics of the study populations for the two study periods are shown in Table 1. There were no statistically significant differences (P > 0.05; for all comparisons). Post-accreditation, the mean (SD) length of stay was 35.3 ± 18.5 days and the range was 3–113 days. Whereas in the pre-accreditation period, the mean (SD) length of stay was 41.1 ± 29.5 days and the range was 1–167 days. The difference was statistically significant (P = 0.026), as shown in Table 2. Further analysis has been performed after excluding the very long LOS of 167 days in the before-accreditation group for its potential outliner effect, and the difference remained statistically significant P < 0.042.

**Table 1:** Comparison between the demographic and clinical characteristics of pre- and post-accreditation patients

Variable	Study period		P value*
	Pre-accreditation	Post-accreditation	
	n = 177 (%)	n = 182 (%)	
Sex			0.999
Male	82 (46.3)	84 (46.2)	
Female	95 (53.7)	98 (53.8)	
Age (years)			0.590
<25	50 (28.2)	59 (32.4)	
25–50	104 (58.8)	104 (57.1)	
>50	23 (13.0)	19 (10.5)	
Marital status			0.352
Single	98 (55.4)	112 (61.5)	
Divorced/separated	20 (11.3)	22 (12.1)	
Married	59 (33.3)	48 (26.4)	
Diagnosis			0.089
Organic disorders	6 (3.4)	11 (6.0)	
Psychotic disorders	78 (44.1)	61 (33.5)	
Affective disorders	73 (41.2)	94 (51.6)	
Others disorders	20 (11.3)	16 (8.8)	

* Level of statistical significance is 5 %

**Table 2:** Comparison of length of hospital stay before and after accreditation

LOS (in days)	Before accreditation (n = 177)	After accreditation (n = 182)	P value
Mean–SD (n)	41.1 ± 29.5	35.3 ± 18.5 (182)	0.026*
Range	1–167	3–113	
Median	34	34	

* Statistically significant at 5 % level of significance

The practice of weekly objective assessment of symptoms severity with the Brief Psychiatric Rating Scale (BPRS) increased significantly from 17.5 % of patients during the pre-accreditation period to 44.5 % of patients during the post-accreditation period (P < 0.001).

## Discussion

This study has a specific focus on the effect of accreditation on the LOS rather than investigating the predictive factors of LOS or delayed discharges “bed-blockers”.

The accreditation process encourages validating the success of good clinical practices. To ensure that accreditation presents a high quality of care, evaluation should be based on the outcome indicators of patients’ care. The length of hospital stay has repeatedly been used as a sensitive quality indicator of efficiency for inpatient care and hospital performance [18, 19].

In this study, there was no significant difference between diagnostic groups in pre- and post-accreditation periods. Research has revealed controversial results regarding the influence of psychiatric diagnosis on LOS, and it is the severity of the psychopathology that has the significant effect on LOS independent of the diagnostic category [20–23].

In agreement with previous research findings [1–4], the results of this study support a positive effect of accreditation programs on clinical outcomes. The significant decline in the mean LOS post-accreditation can be explained by the cumulative effect of several practical factors. First, the practice of rapid evaluation and documentation of management plan within seven working days of admission. Second, provision of multidisciplinary intervention with the involvement of caregivers during patients’ psychiatric hospitalizations. A psychologist, a social worker, and a clinical pharmacist were enrolled in the psychiatric patient rounds. Third, enhancement of team communication through accreditation, which is consistent with a previous study describing the work of the Canadian Council on Health Services Accreditation [24]. Fourth, improvement of the objective assessment of symptoms severity through the Brief Psychiatric Rating Scale (BPRS), which articulates with the discharge criteria and plan.

Previous studies revealed that discharge planning reduced LOS and planned discharge with shorter psychiatric hospital stays can be as therapeutically beneficial as longer ones [21, 25, 26]. The length of hospital stay is usually determined by physicians involved in the patient’s care and driven by clinical need. Lack of suitable alternatives, such as community support and long-term rehabilitation beds, may delay discharge from acute psychiatry wards. However, a prolonged LOS may not necessarily be a delayed discharge if the patient continues to require hospital treatment on clinical grounds. It should be emphasized that reducing LOS does not mean premature discharge, which may create pressure on emergency departments and cause more psychiatric morbidity in the long term.

The present study has some limitations that should be addressed. It relied on retrospective data collection and represented data for only one hospital with an inherent selection bias. The study has not investigated the stability of clinical improvement after discharge and the consequences of shorter LOS. To do this, research would have to follow patients for a substantial time after discharge to assess readmission rates and level of functioning. Nevertheless, the results of the study may fill a research gap in understanding LOS in psychiatric inpatients and provide a view regarding accreditation impact on mental health practice in the Saudi context.

Further multicenter studies are required to support the finding of this study, to identify the modifiable determinants of psychiatric LOS and to compare short term with long term hospitalization outcome concerning level of functioning, readmission rates, and stability of clinical improvement after discharge.

## Conclusion

Hospital accreditation, by reducing excess LOS, contributes to improving the quality of psychiatric inpatient care and access to psychiatric beds.

## Abbreviations

LOS: length of stay
ACI: accreditation Canada international
SPSS: Statistical Package for the Social Science
SD: standard deviation
BPRS: brief psychiatric rating scale
KKUH: King Khalid University Hospital
KSU: King Saud University
MDT: multi-disciplinary team
